# Currently Available Treatment Modalities for Uterine Fibroids

**DOI:** 10.3390/medicina60060868

**Published:** 2024-05-26

**Authors:** Jelena Micić, Maja Macura, Mladen Andjić, Katarina Ivanović, Jelena Dotlić, Dušan D. Micić, Vladimir Arsenijević, Jelena Stojnić, Jovan Bila, Sandra Babić, Una Šljivančanin, Danka Mostić Stanišić, Milan Dokić

**Affiliations:** 1 Clinic of Gynecology and Obstetrics, University Clinical Center of Serbia, 11000 Belgrade, Serbia; jdmicic@yahoo.com (J.M.); maja_macura@live.com (M.M.); andjicmladen94@gmail.com (M.A.); ikatarina.1996@gmail.com (K.I.); drenadot@gmail.com (J.D.); ivfjelena@gmail.com (J.S.); bilamsj@gmail.com (J.B.); sandradrmilic@yahoo.com (S.B.); una645652@yahoo.com (U.Š.); mosticd@gmail.com (D.M.S.); 2 Faculty of Medicine, University of Belgrade, 11000 Belgrade, Serbia; ducamicic@yahoo.com (D.D.M.); vlada.dr.ars@gmail.com (V.A.); 3 Clinic of Emergency Surgery, Emergency Center, University Clinical Center of Serbia, 11000 Belgrade, Serbia

**Keywords:** myoma, management, medicaments, surgery, minimally invasive procedures

## Abstract

Uterine fibroids (leiomyomas and myomas) are the most common benign gynecological condition in patients presenting with abnormal uterine bleeding, pelvic masses causing pressure or pain, infertility and obstetric complications. Almost a third of women with fibroids need treatment due to symptoms. Objectives: In this review we present all currently available treatment modalities for uterine fibroids. Methods: An extensive search for the available data regarding surgical, medical and other treatment options for uterine fibroids was conducted. Review: Nowadays, treatment for fibroids is intended to control symptoms while preserving future fertility. The choice of treatment depends on the patient’s age and fertility and the number, size and location of the fibroids. Current management strategies mainly involve surgical interventions (hysterectomy and myomectomy hysteroscopy, laparoscopy or laparotomy). Other surgical and non-surgical minimally invasive techniques include interventions performed under radiologic or ultrasound guidance (uterine artery embolization and occlusion, myolysis, magnetic resonance-guided focused ultrasound surgery, radiofrequency ablation of fibroids and endometrial ablation). Medical treatment options for fibroids are still restricted and available medications (progestogens, combined oral contraceptives andgonadotropin-releasing hormone agonists and antagonists) are generally used for short-term treatment of fibroid-induced bleeding. Recently, it was shown that SPRMs could be administered intermittently long-term with good results on bleeding and fibroid size reduction. Novel medical treatments are still under investigation but with promising results. Conclusions: Treatment of fibroids must be individualized based on the presence and severity of symptoms and the patient’s desire for definitive treatment or fertility preservation.

## 1. Introduction

Uterine smooth-muscle tumors (leiomyomas or myomas), with a prevalence rate of up to 70%, are the most common benign uterine tumors in women during their reproductive years [1]. Fibroids are diagnosed in women of all ages but are most commonly found in women aged 35–50 years. They are monoclonal tumors of uterine smooth muscle. Some of the known factors that influence the development of uterine fibroids are genetics and race; reproductive and hormonal disturbances and obesity and vitamin D deficiency [2]. Although they are benign and can be asymptomatic, around 30% of fibroids cause profuse menstrual bleeding, irregular uterine bleeding, dysmenorrhea, pelvic discomfort and even pain due to pressure on adjacent organs and structures as well as obstetric complications such as infertility, recurrent abortions or preterm labor [3]. The currently adopted classification of fibroids was proposed by FIGO (The International Federation of Gynecology and Obstetrics), and it describes eight types of fibroids and a hybrid class in which two types of fibroids may be present in the same patient (Table 1) [4].

A wide range of therapeutic options is currently available for uterine fibroids (medicamentous and surgical), but their treatment remains an ongoing dilemma for many practitioners. Women generally attempt to avoid surgical treatment because of the potential risks associated with it [5]. Medical and minimally invasive procedures are currently preferred by both patients and gynecologists for fertility preservation. However, surgical intervention sometimes has to be the primary treatment choice [6,7].

Therefore, bearing in mind the significant prevalence and diverse etiology of uterine fibroids, in this review, we present different medicamentous, classic surgical and minimally invasive procedures performed under radiologic or ultrasound guidance to provide more insight into different more contemporary treatment options for uterine fibroids.

## 2. Methods

An extensive search of the available data regarding surgical, medical and other treatment options for uterine fibroids was conducted. The search included Scopus, MED-LINE and PubMed, encompassing studies from the previous 23 years, from 2000 until the end of 2023. The keywords and MeSH ID (if available) that were used, alone or in combinations, were as follows: ‘uterine fibroid’ (MeSH ID: D007889), ‘myoma’ (MeSH ID: D009214), ‘uterine fibroid/myoma treatment/management’, ‘uterine fibroid/myoma surgery’, ‘uterine fibroid/myoma medications’, ‘uterine fibroid/myoma minimally invasive procedures/interventions/therapies’ and ‘uterine fibroid/myoma interventional radiology procedures’. Titles and abstracts of studies retrieved using the search strategy, and those from additional sources, were screened independently by two authors to identify those that met our objective. The full texts of these potentially suitable articles were downloaded and assessed for eligibility by two other team members. Any disagreements between them were resolved through discussion with a third collaborator.

Peer-reviewed publications written in English were included in this review. Two authors independently extracted data from articles using a standard form to ensure consistency. The results of the research have been divided into different sections and subsections to illustrate what has been reported regarding the investigated topic.

This work has some limitations. First, only English-language papers were included. Second, the included studies may vary in terms of the quality, study design and outcomes assessed. Therefore, given the heterogeneous nature of the review, only a narrative synthesis was possible.

## 3. Results and Discussion

Treatment options for uterine fibroids include medicamentous, interventional radiology and surgical options. Generally, it is suggested that the treatment of fibroids begins with medicamentous and minimally invasive treatments before surgery [5,8].

I. Medicamentous Treatment of Uterine Fibroids

The medicamentous management of fibroids is typically reserved for patients with heavy menstrual bleeding and/or pelvic pain who wish to preserve their fertility. For such patients, the optimal treatment opportunity involves the control and stabilization of hormone levels in fibroid cells with different medications. In addition, medicamentous treatment can serve as a presurgical adjuvant to decrease fibroid mass [9,10]. Medicamentous therapy yields effective results, and symptoms usually improve after one year of treatment. On the other hand, medicamentous therapy for fibroids does not have long-term effects. Therefore, the medicamentous management of fibroids is currently limited, as no pharmacological agents can be used in continuity long-term due to potential side effects [11]. Estrogen has been well-recognized for some time as the main hormone involved in the development and growth of uterine fibroids. Previous studies have suggested that progesterone and its corresponding receptors also play a significant role in fibroid evolution. Progesterone has both proliferative and antiproliferative effects that are still being investigated. Therefore, these two hormones and their receptors are the central target for medicamentous fibroid therapy [10].

I a. Combined Oral Contraceptives (COCs)

In the past, it was believed that estrogens and progestogens from COCs could stimulate the fibroid’s growth. However, investigations showed that they can be beneficial for women with abnormal uterine bleeding with and without fibroids. COCs act by downregulating sex/hormone production, triggering suppressive effects on endometrial proliferation [12]. The main advantages of COCs are their accessibility, oral administration and low cost. Conversely, numerous studies have shown that COCs do not diminish uterine fibroid volume or uterine size. Moreover, they have minimal effects on other fibroid-related symptoms, making their use for fibroid treatment limited [13].

I b. Levonorgestrel-Releasing Intrauterine System

The levonorgestrel-releasing intrauterine system is a contraceptive method that is also ideal for the treatment of heavy menstrual bleeding in patients who have contraindications for COCs. In addition, it has long-term effects and can be used for up to five years. Levonorgestrel is released into the endometrium where it suppresses proliferation, leading to atrophy and amenorrhea. It also has reduced systemic adverse effects compared to COCs [14].

On the other hand, its use in women who have myoma-induced heavy menstrual bleeding is limited. While the rate of intrauterine system expulsion is generally low (0–3%), in women with fibroids, the expulsion rate is up to 20%. Therefore, use is not recommended for women who have submucosal fibroids [15].

I c. Selective Estrogen Receptor Modulators (Raloxifene)

The efficacy of Raloxifene for the treatment of uterine smooth-muscle tumors or their symptoms has still been insufficiently researched. In previous research, after Raloxifene with a gonadotropin-releasing hormone (GnRH) analogue was administered for six cycles of 28 days in premenopausal women, a significant reduction in the size of fibroids was registered. However, no difference was detected in fibroid-related symptoms. Furthermore, a possible risk of venous thrombosis with high doses of Raloxifene remains a concern [16].

I d. Aromatase Inhibitors and Androgenic Steroids (Danazol and Gestrinone)

Danazol and Gestrinone are aromatase inhibitors and androgenic steroids that are sometimes effective in diminishing fibroid-associated symptoms. Unlike Danazol, Gestrinone can even reduce the size of fibroid mass in perimenopausal women [17]. Danazol inhibits ovarian estrogen production via pituitary gonadotropin secretion inhibition. It has been proven in various studies to reduce the symptoms associated with fibroids, but it has not been proven to exert effects on the fibroid size. Androgenic side effects such as weight gain, muscle cramps, hot flashes, mood changes, depression, acne and hirsutism are commonly reported during the use of androgenic steroids [18].

I e. Nonsteroidal Anti-Inflammatory Drugs and Antifibrinolytic Agents (Tranexamic Acid)

Although these agents are less effective than other medications, nonsteroidal anti-inflammatory drugs can, to some extent, reduce symptoms of pelvic pain and heavy menstrual bleeding associated with uterine fibroids. Tranexamic acid, an antifibrinolytic agent, is very effective in the management of fibroid-induced heavy menstrual bleeding [19,20].

I f. Progestogens

High-dose oral progestogens lead to endometrium decidualization and are frequently used for the short-term management of heavy menstrual bleeding [14]. Some investigations have also confirmed the positive effects of progestogens on reductions in fibroid size and volume. However, high-dose progestogens, either as a monotherapy or in combination with gonadotropin-releasing hormone (GnRH) agonists, cannot be used for long-term treatment because, after some time, this therapy itself might cause spotting and even increased bleeding due to changes in endometrial vasculature [21].

I g. Selective Progesterone Receptor Modulators (SPRM)

Biochemically, SPRMS are ligands that have selective progesterone agonist, antagonist or dual activity on progesterone receptors [22,23]. Their effects are better within fibroids in which the expression of progesterone receptors is increased. They include several medications, of which the most commonly used are mifepristone, ulipristal acetate (for short-term therapy) and vilaprisan [24,25]. SPRMs cause apoptosis in tumor cells, followed by the downregulation of the proliferation of cells involved in collagen synthesis, with subsequent reductions in the extracellular matrix [26]. Thus, SPRMs have been shown to diminish uterine fibroid volume by 17–57% and to reduce uterine mass by 9–53% [24,27]. Moreover, with this therapy, the recurrence of fibroids is reduced for up to six months even after the cessation of the use of SPRMs [28,29]. In women with heavy menstrual bleeding and fibroids, the precise mechanisms of SPRM action involve effects on uterine blood flow, endometrial vessels and ovulation, as well as direct progesterone receptor-mediated effects on uterine fibroid cells, including proapoptotic and antiproliferative effects [30]. In addition, SPRMs do not affect bone mineral density, placing them in a highly favorable category of medical options for fibroid treatment [22,25].

I h. Gonadotropin-Releasing Hormone (GnRH) Agonists and Antagonists

GnRH agonists, when administered, rapidly stimulate the pituitary gonadotrophs to secrete follicle-stimulating hormone (FSH) and luteinizing hormone (LH). Via a feedback loop, they subsequently downregulate receptors and inhibit the pituitary–gonadal axis. Gonadotropin secretion is amplified by the flare-up effect of GnRH, but sustained GnRH secretions cause the downregulation of GnRH receptors, which in turn causes decreases in LH and FSH [31].

GnRH antagonists suppress the secretion of FSH and LH by blocking pituitary GnRH receptors. GnRH antagonists prevent the agonists’ first stimulatory phase, which causes them to reduce pituitary gonadotropins through GnRH receptor competition. Because of their quick action and the avoidance of a gonadotropin flare effect, symptoms are promptly relieved [32].

Therapy with either GnRH agonists or antagonists puts patients in a pseudo-menopausal state and reduces the size of fibroids and associated symptoms. The suppression of the gonadal axis results in the hypoestrogenic effect, which improves menstrual bleeding and reduces uterine fibroid size in about 3 to 4 weeks after the initiation of treatment [13,31].

Therapy with GnRH agonists is recommended in cases involving large uterine fibroids (>10 cm). Data from the literature indicate that GnRH antagonists can be administered for a longer time to provide persistent gonadal suppression. Both GnRH agonists and antagonists are particularly used as adjuvant therapy before laparoscopic myomectomy to reduce the operative time and blood loss during surgery. Menopausal symptoms such as hot flashes, mood swings and vaginal dryness are the most common side effects that may limit their use. The most severe side effect is decreased bone mineral density [13,32,33].

I i. Nonpeptide Oral Gonadotropin-Releasing Hormone Antagonists

Nonpeptide is a short-acting orally active GnRH antagonist. It reversibly blocks receptor signaling, which suppresses LH and FSH in a dose-dependent manner and consequently leads to the suppression of ovarian sex steroids [34]. Unlike GnRH agonists, which cause hormonal suppression by desensitizing GnRH-R, the effects of nonpeptide have a faster onset and cessation. In that manner, therapy with oral GnRH antagonists overcomes the difficulties and costs associated with injectable GnRH antagonists [12]. Nonpeptide has been found to be effective in relation to fibroid-induced bleeding, the reduction in other symptoms and quality-of-life improvements. Nonpeptide is effective and safe for short-term use. The main drawbacks are dose-dependent bone mineral density reduction, low-density lipoprotein serum level increase and hypoestrogenic adverse effects, such as hot flashes [35].

I j. Statins

Recent research has demonstrated that uterine fibroids may be treated using antihyperlipidemic medication statins [36]. Statins cause apoptosis and suppress the growth of fibroid cells via calcium-dependent pathways. Additionally, low doses of statins can prevent extracellular matrix protein production in myomas [37,38].

II. Interventional Radiology Procedures

Minimally invasive treatments for uterine smooth-muscle tumors are designed with the aim to preserve the fertility of women. They can also be a promising alternative therapeutic option for women who are poor surgical candidates. Such treatments include uterine artery embolization (UAE), magnetic resonance-guided focused ultrasound (MRgFUS) and radiofrequency ablation (RFA). All of these procedures are correlated with satisfactory clinical outcomes [39]. However, in patients with adenomyosis, endometriosis and/or ovarian cysts, their success is debatable. Submucosal fibroids (type 0 and type 1) and pedunculated subserosal fibroids are not candidates for any of these techniques because the risk of necrosis with expulsion is high [40]. Notably, no well-designed comparative studies have examined pregnancy outcomes and successes between different techniques. Additionally, the necrosis width effect (coagulative or ischemic) on the uterus walls and surrounding tissue and the consequent effect of the degenerated fibroids on implantation, placentation and contractility are still unknown [6,22].

II a. Uterine Artery Embolization

Uterine artery embolization (UAE) is an interventional radiologic technique with an excellent clinical success rate that results in similar quality of life compared to the surgical management of fibroids, wherein an occlusive agent is used to block the uterine arteries or individual branches that feed a fibroid [41,42]. As the uterus receives accessory blood flow, normal uterine tissue heals from the ischemic shock. In contrast, fibroids are unambiguously damaged, which results in ischemic damage with necrosis and finally an irreparable reduction in fibroid size [43,44].

The most relevant issues associated with the technique are the impact of UAE on reproductive function (loss of ovarian function, radiation exposure and the possibility of subsequent hysterectomy in case of complications) as well as complications of pregnancy after UAE (attachment anomalies of the placenta, fetoplacental insufficiency leading to fetal growth restrictions and premature birth) [45]. Despite the low invasiveness of such treatment, the average hospital stay after UAE is 4 to 6 days. UAE complications include pain, vaginal discharge and post-embolization syndrome, which consists of mild fever, fatigue, nausea, vomiting, myalgia and leukocytosis. Despite the presence of a significant number of registered reports of healthy pregnancies without complications after the UAE, currently, UAE is not considered a method of choice for symptomatic fibroids for women planning pregnancy [46].

II b. Uterine Artery Occlusion (Via Laparoscopy or a Vaginally Placed Clamp)

Uterine artery occlusion is a technique in which a clamp is placed on the uterine artery either via laparoscopy or vaginally [47]. This technique has some advantages over the embolization of uterine arteries, as it enables a direct laparoscopic assessment of the pelvis and abdomen and does not require the introduction of foreign material [48]. The data in the literature suggest that, after occlusion, menstrual blood loss decreases by 50% and the size of prominent fibroids decreases by 36% six months after treatment. Additionally, compared to uterine artery embolization, postoperative pain and painkiller usage have been found to increase [49]. However, when compared with UAE, some of the disadvantages include lower mean uterine volume reductions (33% vs. 51%) and the higher recurrence of symptoms (48% vs. 17%) [50].

II c. Magnetic Resonance-Guided Focused Ultrasound

MRI-guided focused ultrasound surgery is a noninvasive method that uses ultrasound thermal ablation to provoke coagulation necrosis affecting the fibroids individually [51]. Magnetic resonance imaging provides a higher resolution for the visualization of anatomic structures and enables real-time thermal monitoring to improve tissue destruction. MRI with gadolinium is used to detect the width and boundaries of tissue devascularization [52]. The major advantage over UAE is the ability to target fibroids individually, while with UAE, ischemic necrosis appears throughout the entire uterus [53]. Moreover, the beneficial effect of the procedure is that there is no risk of ovarian failure, whereby ovarian tissue stays intact. Additionally, the elimination of all tumor cells is not necessary. It is sufficient to coagulate separate points inside the fibroid to provoke fibroid reduction and improve symptoms [54].

Limiting factors for this treatment are the size, vascularity and accessibility of fibroids, in conjunction with the fact that the procedure can be lengthy and costly. In addition, the fibroid cannot be treated via this method if the bladder, colon or nerves obstruct the ultrasound waves’ passage [51]. Furthermore, duration of the procedure for safety concerns should not extend more than three hours, which limits early treatment completion and success [53]. Finally, drawbacks of the technique include prolonged and heavy menstruation after treatment and occasional pain in the lumbar areas because of the heating of the sciatic nerve roots. Nevertheless, about 80% of women exhibit improvements in their symptoms, while not more than a quarter of women need another round of the procedure after 4 years [52,54].

II d. Radiofrequency Ablation

Radiofrequency ablation causes coagulative necrosis by applying monopolar energy. When a fibroid or other tissue is exposed to high-frequency alternating current (in the radiofrequency range of 3 kHz to 300 GHz), tissue ions oscillate, producing heat that leads to protein denaturation and cell death [55]. Radiofrequency can be applied via laparoscopic, hysteroscopic or transvaginal routes. It is generally safe and effective, with few severe complications (bleeding, uterine perforation and intestinal perforation). The major risk factor for complications is large size of the fibroid. Therefore, some experts have suggested performing the procedure on less than three fibroids on one occasion and only on fibroids under 110 cc in volume and 5 cm in diameter, classified as FIGO 0–4 type, leaving a safe distance to the serous layer of at least 1 cm [56,57].

II e. Myolysis

Myolysis or myoma coagulation involves the thermal, radiofrequency, laser or cryoablation ablation of fibroid tissue through hysteroscopic or laparoscopic routes. Myolysis can significantly reduce menstrual blood loss and fibroid size [58]. It is a safe and effective therapeutic option associated with shorter hospitalization and reduced intraoperative blood loss in comparison to laparoscopic myomectomy. Nevertheless, data regarding the subsequent outcome of pregnancies after myolysis are scarce. Therefore, hysteroscopic laser myolysis is indicated only for FIGO type 1 or 2 fibroids in women who are not planning future pregnancies [29].

II f. Endometrial Ablation

Endometrial ablation, either alone or with hysteroscopic myomectomy, uses hot, cold or mechanical means to ablate the endometrium and reduce menstrual bleeding in the management of fibroids. Previous studies have indicated that more than 90% of women who had hysteroscopic myoma resection in combination with endometrial ablation did not need surgery after a 9-year follow-up, illustrating the long-term efficacy of endometrial ablation in the treatment of fibroid-induced bleeding. However, since this procedure does not affect intramural and subserosal fibroids, pressure symptoms associated with larger fibroids cannot be addressed. Subsequent pregnancies after endometrial ablation may involve the risk of ectopic pregnancy, prematurity or abnormal placentation [59,60,61].

III. Surgical treatment of uterine fibroids

Myomectomy is a surgical treatment in which the fibroids are removed and the uterus is then reconstructed. Therefore, myomectomy remains the standard treatment for women who are planning future pregnancies.

Myomectomy improves symptoms in up to 80% of cases but is associated with about a 27% risk of recurrence if one fibroid is removed and a risk level greater than 50% in the case of multiple fibroids. The literature suggests that myomectomy can decrease the rate of miscarriage in patients with myomas that destroy the cavity of the uterus and increase postoperative pregnancy rates to more than 50% [1].

Myomectomies generally have a low rate of complications (1–5%). The most common complication is blood loss during surgery, while the most severe is thromboembolism [62]. Nowadays, various medications and procedures can decrease blood loss during myomectomy, such as the intra-fibroid infiltration of vasopressin, intravaginal misoprostol or dinoprostone, the use of pro-fibrin/thrombin agents or the use of a bandage/tourniquet around the cervix or infundibulo–pelvic ligaments to compress the uterine blood vessels and reduce the blood supply to the uterus during surgery [63,64]. The single technique which is generally used entails the application of the tourniquet around the cervix to occlude both uterine arteries, while the triple technique involves the occlusion of the ovarian vessels as well. To prevent ischemic injuries, the vessel occlusion should be performed medial to the ovaries and Fallopian tubes. The tourniquet can be a suture, clamp or Foley catheter. Finally, a uterine artery ligation can also be performed [65]. Another significant postoperative complication is the occurrence of scars that lead to the disruption of normal uterine tissue. This is most important during pregnancy due to the increased risk of ruptures caused by stretching and contractions during the delivery [66].

Surgery can be performed via hysteroscopy, laparoscopy, laparotomy or through a vaginal route for nascent myomas. The choice of the surgical treatment depends on the localization, size, type and number of fibroids, the experience of the surgeon and the anesthetic risks [8,67].

III a. Hysteroscopic Myomectomy

Hysteroscopic myomectomy is indicated for submucosal fibroids. According to FIGO, for leiomyomas classification types 0, 1 and 2, a standard procedure is the wire loop resection under direct visual guidance [68]. Fibroids should be located at least 5 mm away from the uterine serosa to prevent uterine perforation. Fibroids less than 4 cm in diameter can be easily resected, while in the case of larger fibroids, hysteroscopy becomes difficult to perform which increases potential complications such as excessive bleeding, uterine wall perforation, etc. [6]. Therefore, all submucosal leiomyomas larger than 3 cm should be treated before surgery with gonadotrophin-releasing hormone agonists (GnRHa) or selective progesterone receptor modulators (SPRM) to decrease the leiomyoma size and make resection possible. On the other hand, in the case of fibroids larger than 4 cm in diameter, a two-step resection with 2- to 3-month intervals should be performed [69,70].

Hysteroscopic myomectomy has a low rate of complications (below 1%). The perioperative risks include excessive liquid absorption, injury of the bladder and bowel and excessive bleeding and uterine perforation, mostly occurring during cervical dilatation [71]. The substances used for uterine distension can also have different risks. If a hypotonic solution is used together with monopolar energy, it may lead to hyponatremia, which can result in neurological complications. When combined with bipolar energy isotonic solution, as in the case of excessive fluid absorption, hypotonic solution may cause a circulatory overload and lead to pulmonary oedema [72]. For that reason, fluid inflow and outflow should be carefully monitored. Major postoperative complications include the formation of intrauterine adhesions. To prevent adhesions, applying anti-adhesion gel or intrauterine devices with oxidized regenerated cellulose and silicon immediately after surgical intervention is suggested [73,74,75].

Hysteroscopic myomectomy can be performed with several techniques according to myoma type and size. For the pedunculated fibroids, the base can be cut and the fibroid extracted with forceps, while for the intramural fibroids, the usual approach is the slicing technique. During myomectomy, the pseudocapsule and the surrounding healthy myometrium should be preserved to enable uterine regeneration. Therefore, the myomectomy is completed when the fasciculate fibers of the myometrium are visualized. In the case of large fibroids type 1–3, a two-step procedure can be done. During the first step, a portion of the fibroma protruding into the uterine cavity is resected. This causes the residual intramural part of the fibroid to descend into the uterine cavity making it accessible for excision during the second-step hysteroscopy [4,22].

Hysteroscopic myomectomy recently showed notable improvements that continue to spread with new devices and techniques. Outstanding results of this quick, cost-effective procedure have been noted in relation to irregular bleeding and fertility improvement. According to the literature, the hysteroscopic surgical resection of submucosal fibroids increases pregnancy rates up to 75% (usually around 50%). However, the success of the procedure must always be individualized based on patient risk factors, including the number, type, size and location of fibroids, as well as the purpose of the procedure [68,69,70].

III b. Laparoscopic Myomectomy

Laparoscopic myomectomy is the minimally invasive treatment approach of choice for the fertility-sparing management of the most symptomatic intramural and subserosal smooth-muscle tumors (FIGO type 3 and 7); this approach decreases the level of postoperative pain, contributes to lower rates of postoperative fever, lowers blood loss and reduces the necessity of blood transfusions. It also contributes to shorter hospitalization time and a rapid recovery time, with a quicker return to normal daily living activities compared to open surgical methods. Laparoscopic myomectomy is a safe and efficient surgical procedure, for which complication rates are lower than 10% [76].

There are no standardized guidelines about the criteria for a laparoscopic approach to myomectomy, and such guidelines have been quite variable, based on the number, size and position of fibroids. The evaluation of fibroids via ultrasound (and, if it is needed, via magnetic resonance imaging) is of significance for correct pre- and intraoperative surgical planning to ensure complete excision during the procedure, due to the inability to directly palpate the fibroids during laparoscopy [77]. Some authors suggest that the laparoscopic approach should be avoided in cases involving more than four fibroids in different sites of the uterus requiring numerous incisions, in cases of large fibroids (larger than 10–12 cm) or if fibroids are located in an intraligamental location [67]. Others have recommended that for the laparoscopic approach, a uterus has to be smaller than the size of 16 gestational weeks, with less than five fibroids, whereby no fibroid should be greater than 15 cm [78]. A diameter of the člargers myome of 12 cm or more significantly increases the occurrence of surgical complications. College National des Gynecologues—Obstetriciens Francais has stated that if a leiomyoma’s diameter is less than 8 cm and only three of them are present, a laparoscopic surgery could be performed [79]. In the case of multiple myomas, laparoscopic myomectomy can be performed in several stages. Large fibroids can be safely and effectively treated combining uterine artery embolization as a first step and laparoscopic myomectomy (performed either on the same day or couple of days later) as the second therapeutic step [67,78].

The newest laparoscopic innovation is laparo-endoscopic single-site surgery or single-port laparoscopy. However, it requires more operative time, and the data about its benefits in comparison with standard laparoscopic surgery are currently limited [80].

Several complications have been registered in relation to laparoscopic myomectomy. Previous studies have suggested that an increased risk of complications is present in case of multiple fibroids, large fibroids and in fibroids with an intraligamental location [81]. Additionally, the risk of conversion to hysterectomy is increased in cases when fibroids involve the cervix, broad ligaments and uterine cornua. Complications of laparoscopic myomectomies include longer operative times, especially for larger fibroids that require morcellation [45,82]. Although morcellation (cutting large masses of tissue into smaller pieces) can be performed manually, a device called a power morcellator is commonly used. However, using a power morcellator increases the risk of the dissemination of the removed tissue into the abdominal cavity which can, although rarely, lead to the occurrence of pelvic adenomyosis and parasitic leiomyomas. To prevent this complication, morcellation in the endo-bag has been suggested along with extensive peritoneal lavage [22,83]. However, the most concerning complication involves pregnancy-related uterine rupture. Although it is rare, uterine rupture can be lethal, and although it is most frequent during pregnancy, its occurrence and risk are unpredictable. Therefore, when laparoscopic myomectomy is considered in reproductive-aged patients, this hazard should not be overlooked [84].

III c. Laparoscopically Assisted Myomectomy

Laparoscopically assisted myomectomy offers an updated approach between laparoscopic and open abdominal surgery [85]. After the laparoscopic entrance into the abdomen and the inspection of the abdominal-pelvic cavity, a suprapubic mini-incision (typically at the level of a standard Pfannenstiel incision) is made. A self-retaining retractor is placed for an adequate exposure through the mini-laparotomy site. In that manner, the position of the uterus can be brought up to the level of the anterior abdominal wall or through the abdominal incision, allowing a myomectomy across a traditional open technique [86]. This approach should be considered for large fibroids in which major uterus reconstruction or multilayer closure is predicted. Laparoscopically assisted myomectomy is especially useful in cases with deep intramural smooth-muscle tumors where the surgeon is able to utilize palpation for advanced intraoperative surgical planning. Lastly, it enables improved visualization and exposure in situations with difficult hemostasis. In general, postoperative care and considerations for laparoscopically assisted myomectomy procedures mirror those of laparoscopic myomectomy [49,80].

In addition, myomectomy of multiple or large intramural fibroids as well as fibroids of the hybrid type can also be performed through a mixed laparoscopic and hysteroscopic approach [1].

III d. Robotically Assisted Myomectomy

Robotically assisted myomectomy is a relatively new minimally invasive approach. The abdominal approach involves a central 8 or 12 mm camera port (usually 8–10 cm above the target anatomy) along with two to three ancillary 8 mm ports for the assistant robotic arms and an optional assistant side port. The steps and technical considerations for myomectomy and specimen extraction are otherwise the same as those for the conventional laparoscopic approach [87,88].

Though in this approach tactile feedback is lost, robotically assisted surgery provides several advantages over conventional laparoscopic myomectomy. It allows for a three-dimensional stereoscopic view, greater dexterity with seven degrees of freedom in each of the jointed instruments and the mitigation of hand tremors, which can facilitate fibroid dissection and multilayer suturing. As such, the robotically assisted approach is safe and effective and therefore should be considered in more technically challenging cases, such as those with particularly large bulky fibroids, tumors involving the cervix and lower uterine segment or extending into the pelvic sidewall or extensive pelvic adhesive disease [89,90].

Overall, robotically assisted myomectomy confers similar patient care benefits in comparison to laparoscopic myomectomy. The common disadvantage of robotic surgery is the financial cost, including higher hospital/professional charges and hospital reimbursement rates [87,89].

III e. Open Myomectomy

Open myomectomy is suitable for the removal of larger fibroids. This technique enables excellent operative field exposure, which is helpful to carefully palpate and inspect the whole uterus for fibroids. It is substantially easier to repair uterine defects and control blood loss during an open myomectomy. An open myomectomy allows the removal of fibroids from the uterus, with the size corresponding to 36 weeks of gestation, with acceptable operative time [91]. This surgery involves myometrial incision, myoma enucleation, hemostasis, re-approximation of the myoma bed and suturing and reconstructing the uterine wall.

On the other hand, the open approach is associated with more postoperative pain, a higher rate of postoperative fever, a longer hospitalization and a longer recovery time; however, it is safer and more efficient in the context of larger and deeper lesions, with more adhesions. Suturing the uterus with an open approach is safer for women who want to give birth after surgery because it bears a smaller risk of uterine rupture [62,92].

Finally, open myomectomy can be performed using a mini-laparotomy, which is a very safe and effective minimally invasive open surgical technique that can enable the same-day discharge of patients [93].

III f. Hysterectomy

A hysterectomy is the last treatment option for symptomatic fibroids. A hysterectomy is a definitive surgical treatment option that eliminates the risk of future recurrence. Approximately one-third of all hysterectomies worldwide are performed due to uterine fibroids. However, it is an advisable option only for women who have finished their reproduction and are content with losing their uterus [94,95].

Hysterectomies can also be performed using vaginal or abdominal (classic open, laparoscopic or robotic) approaches [96]. Vaginal and minimally invasive hysterectomies are recommended whenever possible, as they are associated with faster and better recovery, leading to shorter hospital stays and better patient satisfaction [45].

The vaginal approach is associated with less morbidity than abdominal hysterectomies. Nevertheless, vaginal hysterectomies are usually limited by the size of fibroids and the uterus. Consequently, pretreatment with GnRH analogs has been suggested to facilitate vaginal hysterectomies [19].

Nowadays, laparoscopic hysterectomy is the typical approach for the majority of patients. However, it often necessitates the morcellation of the uterus, although iatrogenic dissemination caused by the function of the morcellator of benign and potentially malignant tissue in undiagnosed leiomyosarcoma has been discussed. Manual or morcellation in the endo-bag is recommended [7,84]. Nevertheless, a uterine volume of more than 13–14 gestational weeks is considered a relative contraindication for laparoscopic hysterectomies. In such cases, a classic open abdominal approach would be the most appropriate [97].

Although an abdominal hysterectomy may reduce the chance of spreading unrecognized malignancy from the uterus to other abdominal structures, it is associated with increased rate of different surgery related complications (blood loss, thromboembolism, abdominal wall wound infection, etc.) when compared with minimally invasive approaches. Therefore, it is indicated as the therapy of choice only for large myoma and in women older than 50 years and/or postmenopausal due to their increased risk for uterine malignancy [98].

## 4. Conclusions

Symptomatic uterine fibroids require surgical and/or medical therapy according to the FIGO classification, severity of symptoms, patient’s age, infertility and wish to preserve fertility. Currently, the usual treatment for fibroids is surgical intervention, such as hysterectomy and/or fertility-sparing myomectomy performed via hysteroscopy, laparoscopy or laparotomy.

Other minimally invasive techniques, such as uterine artery embolization and occlusion, myolysis, magnetic resonance–guided focused ultrasound surgery, radiofrequency ablation of fibroids and endometrial ablation can provide excellent clinical outcomes in the appropriately selected patient but are less often used in everyday practice. Moreover, these procedures are not suggested for very large fibroid and uterus sizes, large numbers of fibroids or submucosal and pedunculated subserosal fibroids because of the risk of necrosis with expulsion. In addition, these procedures are also not recommended if future pregnancy is desired because of the effect of necrosis (coagulative or ischemic) on the uterus caused by these procedures, and the effect of the degenerated fibroids on implantation, placentation and uterine contractility remain unknown.

Medicamentous treatment options for fibroids are restricted, and available medications are generally used for short-term treatment of fibroid-induced bleeding. Progestogens and COCs provide a brief symptom improvement but do not reduce fibroid size. GnRH agonists are highly effective in suppressing bleeding and reducing fibroid size, but because of their side effects, they cannot be used for long periods. Recently, there is growing evidence that SPRMs can be very efficient for uterine fibroids in symptomatic women. Studies showed that they could be administered intermittently long-term with good results on bleeding and fibroid size reduction. Novel medical treatments, although still under investigation, seem to be good preoperative treatment options as well as an alternative to surgical intervention for fertility preservation.

Finally, it should be pointed out that, based on the fact that fibroid development and growth is an interplay between genetic, epigenetic, hormonal, lifestyle and environmental factors, uterine fibroid management should be individualized according to the medical condition and patient symptoms and wishes (Table 2).

## Figures and Tables

**Table 1:** FIGO classification.

Myoma Classification		Explanation
Submucosal group	Type 0	pedunculatedintracavitary
	Type 1	<50% intramural
	Type 2	≥50% intramural
Other group	Type 3	100% intramural; contacts endometrium
	Type 4	intramural
	Type 5	subserosal ≥50% intramural
	Type 6	subserosal <50% intramural
	Type 7	subserosal pedunculated
	Type 8	other, e.g., cervical, parasitic
Hybrid leiomyoma group (impacting both the endometriumand serosa)		submucous and subserous each with less than half of the diameter in the endometrial and peritoneal cavities
		two numbers listed separately separated by a hyphen with the first number indicating the endometrial relationship and the second number indicating the serosal relationship

**Table 2:** Key points for uterine fibroma treatment.

1. Treatment options for uterine fibroids include medicamentous, interventional radiology and surgical options. Generally, it is suggested that treatment of fibroids begins with medicamentous and minimally invasive treatments before surgery. However, surgery (myomectomy or hysterectomy, open or endoscopic) is the most common treatment as medicamentous treatment is restricted and used only short-term while other minimally invasive techniques are not recommended if future pregnancy is desired.
2. Decisions about uterine fibroma treatment must balance the risks and benefits and should be individually based on patients’ medical conditions, symptoms and wishes. Decision should be made between the patient and her obstetrician–gynecologist as well as other health care professionals.

## Data Availability

Data are contained within the article.
